# Assessment of magnitude and spectrum of cardiovascular disease admissions and outcomes in Saint Paul Hospital Millennium Medical College, Addis Ababa: A retrospective study

**DOI:** 10.1371/journal.pone.0267527

**Published:** 2022-12-12

**Authors:** Mekoya D. Mengistu, Henok Benti

**Affiliations:** 1 Department of Physiology, School of Medicine, Addis Ababa University, Addis Ababa, Ethiopia; 2 Department of Internal Medicine, Yekatit-12 Hospital Medical College, Addis Ababa, Ethiopia; 3 Department of Internal Medicine, St. Paul Hospital, Millennium Medical College, Addis Ababa, Ethiopia; University of Campania Luigi Vanvitelli: Universita degli Studi della Campania Luigi Vanvitelli, ITALY

## Abstract

**Background:**

Cardiovascular diseases(CVD) remain the leading cause of death in the world and over 80% of all cardiovascular-related deaths occur in low and middle income countries. Ethiopia is in epidemiologic transition from predominantly infectious diseases to non-communicable diseases and the CVD is a major public health challenge.

**Methods:**

The aim of this study was to assess the magnitude and spectrum of cardiovascular admission and its outcomes among medical patients admitted to both Medical Ward and ICU of St. Paul Teaching Hospital from 1st of Jan 2020 to 1st of Jan 2021.

**Results:**

Out of 1,165 annual medical admissions, the prevalence of cardiovascular diseases(CVD) was 30.3%. About 60%(212) of patients had advanced congestive heart failure of diverse causes. Hypertensive heart disease (HHD) was the next predominant diagnosis (41%(146)), and also the leading cause of cardiac diseases followed by rheumatic valvular heart disease(RVHD) (18%(64)) and Ischemic heart disease (IHD) (12.2%(43)), respectively. Yong age, rural residence and female sex were associated with RVHD(p = 0.001). Stroke also accounted for 20%(70) of CVD admission (hemorrhagic stroke-17% Vs Ischemic stroke-83%). Hypertension was the predominate risk factor for CVD and present in 46.7%(168) of patients. The mean hospital stay was 12days and in-hospital mortality rate was 24.3% with septic shock being the commonest immediate cause of death followed by fatal arrhythmia, brain herniation, and massive PTE.

**Conclusion:**

Cardiovascular diseases were common in the study area causing significant morbidity and mortality. Therefore, comprehensive approach is imperative to timely screen for cardiovascular risk reduction, disease control and complication prevention. Strategies should also be designed to increase public awareness regarding the cardiovascular risk reduction, drug adherence, and possible complications.

## Introduction

The impacts of non-communicable diseases (NCDs) are particularly devastating in poor and vulnerable populations [1]. NCDs currently cause more deaths than all other causes combined and is projected to increase from 38 million in 2012 to 52 million by 2030 [2]. Nearly three quarters of all NCD deaths occur in low- and middle-income countries. NCD deaths will increase by 17% over the next ten years and the greatest increase will be seen in the African region [2]. In Ethiopia, the recent NCD deaths are estimated at around 42% with cardiovascular diseases are the predominant cause of morbidity and mortality [3–5].

Cardiovascular disease remains the leading NCDs related cause of death in the world. Approximately 80% of all cardiovascular-related deaths occur in low and middle income countries and at a younger age in comparison to high-income countries [6]. Africa is home to for over 1 billion people, and is a major contributor to the global burden of CVD [7,8]. In 2013, an estimated 1 million deaths were attributable to CVD in sub-Saharan Africa alone [7,9]. CVD-related deaths contributed to 38% of all non-communicable disease-related deaths in Africa, reflecting the growing threat of both non-communicable disease and CVD [9]. The understanding of adults about CVD and its risk factors including hypertension and diabetes is low in developing countries. More than half of adults in sub-Saharan Africa have poor knowledge of CVD [10,11]. Similarly, in Ethiopia the knowledge of cardiovascular risk factors among CVD patients was unsatisfactory, and about half of the patients have suboptimal knowledge [12]. In another study, 52% of Ethiopian hypertensive patients on follow up have poor basic knowledge of hypertension, and about 60% of them have poor practice towards control of hypertension [13]. Patient noncompliance is also one of the most difficult challenges in the healthcare sector and therefore, it is vital to enhance patients’ awareness and knowledge about the complications and benefits of cardiovascular medications [14].

A cardiovascular spectrum study in Addis Ababa has revealed the five most common cardiovascular diseases such as valvular heart disease (62%), hypertension (14.7%), cerebrovascular disease (11.5%), congenital heart disease (8.5%), and ischemic heart disease (IHD) (6.8%) [15]. Another outpatient CVD spectrum study also demonstrated that RHD was the common cause of cardiovascular disease followed by HHD and cardiomyopathy [16]. Similar study in the cardiac follow up clinic of Jimma Specialized Hospital showed that rheumatic heart disease was the leading cause of cardiac illness (32.8%), followed by hypertensive heart disease (24.2%), cardiomyopathy(20.2%), arrhythmia(13.5%) and cor-pulmonale(3.8%) [17]. However, there is an increasing trend of hypertensive heart disease in the subsequent periods replacing the place of valvular heart diseases.

A recent outpatient study conducted in Northern Ethiopia revealed that Hypertensive heart disease was the predominant etiologic diagnosis of cardiovascular disease followed by rheumatic heart disease [18]. In this study, hypertensive heart disease has markedly surpassed rheumatic heart disease as the leading heart disease which might be due to the high proportion of hypertension (62.3%) among the CVD patients in the area [18]. In another African studies hypertension was also the main causes of heart failure (21.3%), followed by rheumatic heart diseases (20.1%), cardiomyopathy(16.8%), coronary artery disease(10%), and congenital heart disease (9.8%) [19]. Different studies have demonstrated that much of the population risk of CVD is attributable to modifiable traditional risk factors, including hypertension, diabetes mellitus, dyslipidemia, smoking, lack of physical activity, and psychosocial factors [6,20]. These risk factors account for 61% of CVD deaths globally and alleviating exposure to these risk factors would improve global life expectancy by almost 5 years [21]. In Ethiopia, there is a paucity of data regarding CVDs and their risk factors, making accurate estimation of burden in terms of the morbidity and mortality of CVDs extremely difficult. This study will therefore provide relevant data regarding the recent magnitude as well as spectrum of cardiovascular admission and its outcome among medical patients in St. Paul specialized hospital, Addis Ababa.

## Methods

### Study setting

The study was conducted in the department of internal Medicine at St Paul Hospital Millennium Medical College (SPHMMC), which is one of the biggest tertiary governmental Teaching Hospitals in Addis Ababa, the Capital of Ethiopia and the seat of African Union. The hospital receives follow-up patients, emergency patients and referrals from other Hospitals and health facilities all over the country.

### Study design

An institutional based, retrospective, cross-sectional study was conducted at St Paul Teaching Hospital involving all the cardiovascular admissions in the medical ward and ICU from 1st of January 2020 to 1st of January 2021. Admission and discharge diagnosis was captured from the registry to further retrieve the chart of the patient for detailed review of demographic data, major investigations, co-morbidities, underlying background risk factors and control, admission/discharge diagnosis of cardiovascular diseases, duration of hospital stay and outcomes.

### Study population

The study included all eligible cardiovascular admissions among all the medical patients admitted to the study hospital during the stated period. A total of 353 patients with cardiovascular diagnosis among all the annual medical admissions of 1165 patients were evaluated during the specified study period. The exclusion criteria were patients whose medical records were incomplete or patients who were died before adequate diagnosis was made. A total of 40 patients with CVD diagnosis on the HMIS registry were excluded due to incomplete medical records.

### Data variables for the study

Relevant patient information was retrieved from the HMIS registry as well as medical records. Cardiovascular diseases included diseases that affect the heart and blood vessels. The main blocks of WHO International Classification of Diseases(ICD)-10th version for Mortality and Morbidity Statistics (MMS) (Version: 04 / 2019) was utilized to sort out the final diagnoses [22]. The unit of analysis was the hospital discharge and/or admission, not the patient and therefore, a patients admitted more than once in a year were counted each time as a separate “admission” to the hospital. In situations with more than one cardiovascular diagnosis in the same case, the different disease conditions were counted separately.

### Operational definition

**Cardiovascular diseases** comprise various diseases affecting the heart and blood vessels.

**Hypertensive heart disease (HHD**) was diagnosed in patients with hypertension presenting with symptoms and signs of heart failure, with or without left ventricular (LV) hypertrophy and left atrial enlargement on two-dimensional echocardiography or Doppler evidence of LV diastolic dysfunction in the absence of significant valvular heart disease or regional wall motion abnormality [16]. For those who have no documented 2-dimentional echocardiogrpahy, ECG evidence of left ventricular hypertrophy (LVH) was used.

**Dilated cardiomyopathy (DCMP)** was diagnosed in patients with marked LV dilatation and dysfunction in the absence of significant valvular, structural, or congenital heart disease or arterial hypertension [16].

**Ischaemic heart disease (IHD)** included any of the three entities: 1. Angina pectoris which is short lived, relieved with termination of the provoking factor or rest and had no typical ECG features of infarction, 2. Acute myocardial infarction which was defined by the presence of elevated cardiac biomarker together with acute onset chest pain, and/or typical ECG changes, 3. Prior myocardial infarction including patients who present with or without heart failure in whom echocardiography detected regional wall motion abnormality in the absence of a history of acute coronary syndrome [16]. Pathologic Q waves on ECG were used to predict the possibility of ischaemia as a cause of the regional wall motion abnormality in a dilated left ventricle with reduced ejection fraction.

**Pulmonary heart disease (PHD):** Right heart failure, also known as cor pulmonale due to altered structure and/or function of the right ventricle evidenced by 2-dimensional echocardiography.

**Blood pressure control**: Controlled BP when the mean BP < 140/90 mmHg in hypertensive patients of all ages [23]. Uncontrolled BP when the mean BP ≥ 140/90 mmHg in hypertensive diabetic patients of all ages [23].

**Blood Sugar control**: Good glycemic control when the mean Fasting blood sugar ≤ 130 mg/dL and /or HbA1C <7% and Poor glycemic control when mean Fasting blood sugar >130 mg/dL and/or HbA1C>7% [24].

### Data collection and instrument

In order to ensure the accuracy, completeness, and comparability of data, four senior medical residents were trained to complete data collection format. Data collection was made by the pretested structured check lists to document all the pertinent profiles of the study subjects.

### Data processing and statistical analysis

After checking for completeness, data was coded, entered, and analyzed using SPSS version 20 software. Descriptive statistics was used to calculate rates. Chi-square was used to estimate the associations between selected predictor variables. A p-value < 0.05 was taken as statistically significant.

### Ethical consideration

Ethical clearance was obtained from institutional review board (IRB) of St. Paul Teaching Hospital. Since it was a retrospective study, written consent was waived by the research and ethics committee. Anonymity of the patient profile was upheld.

## Results

### Socio-demographic characteristics of the study population

**Fig 1:**
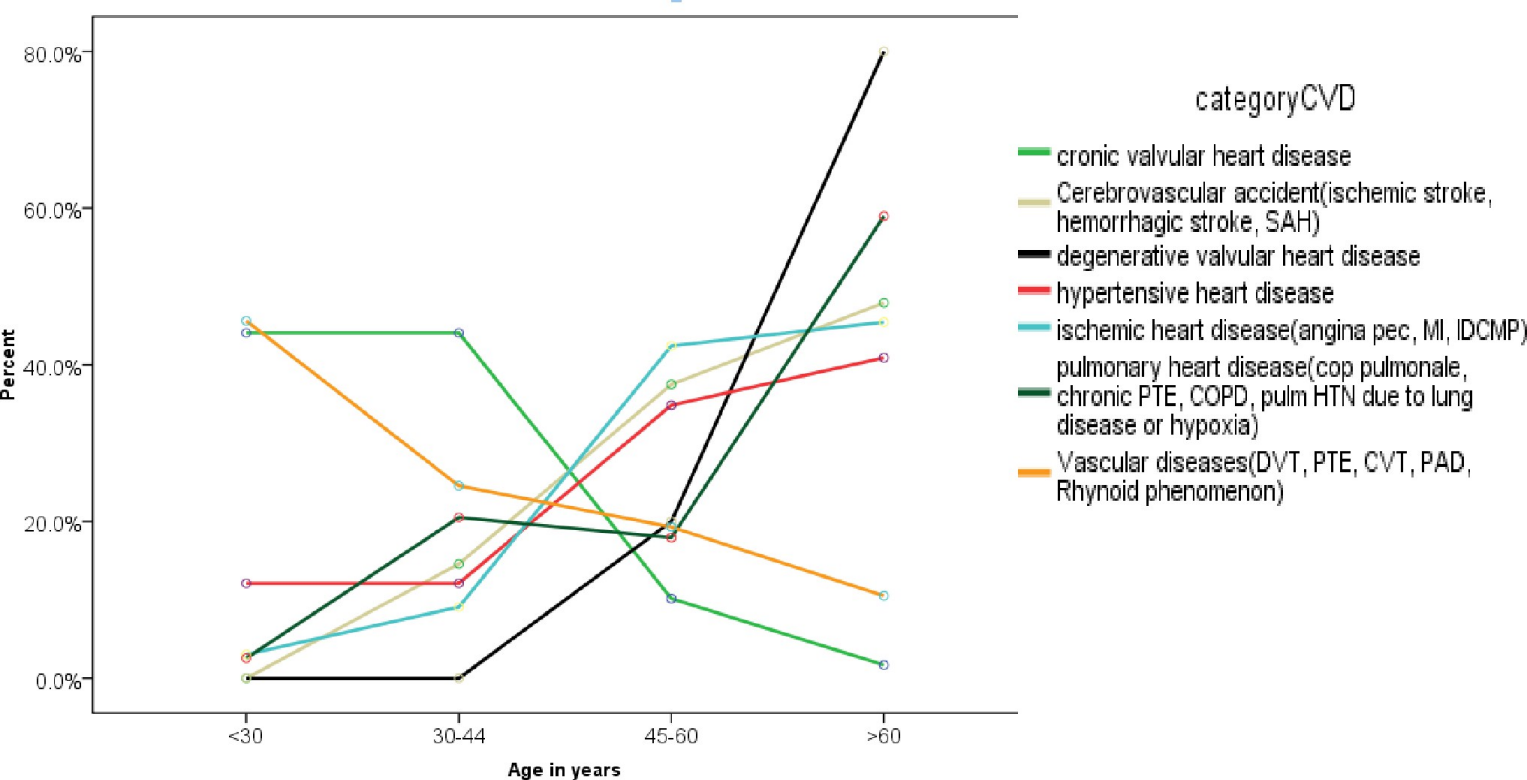
The associations of major cardiovascular diseases with increasing age. p<0.001.

From the annual medical admissions of 1,165 patients to st. Paul Hospital Millennium Medical College from 1^st^ of January 2010 up to 1^st^ of January 2021, the total cardiovascular admissions constituted 30.3%(353). Majority of the study subjects were females (60%(213)). Addis Ababa together with Oromia regional state constituted 90% of total cardiovascular admissions to St. Paul Hospital Millennium Medical College during the study period (Table 1). The minimum age among the cardiovascular admissions was 15years and the maximum age was 86years with the mean and median ages were 48.9 and 50years, respectively. The overall cardiovascular illness increased almost steeply with increasing age(p = 0.001). However, further disease stratification showed that Rheumatic valvular heart diseases(CRVHD) and vascular diseases including deep vein thrombosis (DVT), pulmonary thromboembolism (PTE) and cerebral venous thrombosis (CVT) were more common in the younger age and proportionally decreased as age advances(p = 0.001) (Fig 1).

**Table 1:** Socio-demographic characteristics of cardiovascular admissions.

Socio-demography	categories	Number(n)	Percentage(%)
**Sex**	male	140	39.7%
	female	213	60.3%
**Residence**	Addis Ababa	175	49.6%
	Oromia	143	40.5%
	South	18	5.1%
	Amhara	13	3.7%
	Others	4	1.13%
**Age**	<30	73	20.7%
	30–44	77	21.8%
	45–60	96	27.2%
	>60	107	30.3%

#### Spectrum of cardiovascular diseases and associated risk factors

The spectrum of major cardiovascular diseases was scrutinized according to the ICD-10 classification of diseases [22] and most of the patients had two or more cardiovascular diagnoses. From all cardiovascular admissions, about 60%(212) of patients had advanced congestive heart failure (NYHA class III and class IV) (Table 2). The advanced congestive heart failure (CHF) had multiple underlying etiologies, and also multiple precipitating factors including hypertension(35%(74)), lung infection(pneumonia) (34%(72)), drug discontinuation (25.5%(54)), and arrhythmia (23.6%(50)) predominantly atrial fibrillation with fast ventricular response(Afib with FVR).

**Table 2:** Spectrum of major cardiovascular Diseases in St. Paul Hospital Millennium Medical College.

CVD spectrum	Frequency(n)	Percentage(%)
CHF(NYHA class III and IV)	212	60
HHD	146	41.36
CRVHD	64	18.1
IHD	43	12.2
PHD	40	11.3
DCMP	34	9.6
DVHD	9	2.5
Vascular DiseasesΔ	85	24
CVA or Stroke	70	20
Arrhythmia	68	19.2
Sub-acute bacterial endocarditis(SBE)	10	2.8
Others*	31	8.8

*Thyocardiac disease, Intra-cavitary masses, High output Heart failure, ΔPAD, DVT, PE, CVT.

Hypertensive heart disease (HHD) constituted for 41.4%(146) of the total CVD diagnosis as well as the leading etiologic cause of advanced heart failure. Its prevalence increased with increasing age (p<0.001). HHD was more predominant among urban residents than rural ones ((63%(92) Vs 37%(54), p = 0.001)). Valvular heart diseases (both rheumatic and degenerative valvular heart diseases combined) accounted for about 34.5% of all advanced cardiac failure cases and 20.5% (73) of all the cardiovascular admissions as shown in the Table 2.

Chronic rheumatic valvular heart diseases(CRVHD) was more predominant in patients coming from outside of Addis Ababa compared to residents of Addis Ababa(82.8%(53) Vs 17.1%(11)(p = 0.001). Echocardiography proven isolated MS and MS with MR constituted 26%(19) and 27.5%(20) of all valvular heart diseases, respectively. The proportion of degenerative valvular heart diseases increased with advancing ages (p = 0.01) whereas rheumatic heart diseases decreased as age increased (p = 0.01). Ischemic heart disease (IHD) accounted for 12.2%(43) of all CVD admissions. Its prevalence has significantly increased with age and males had higher proportion than females(p = 0.001). IHD was more common in residents of Addis Ababa than those who came from out of Addis Ababa but the difference was not significant (p = 0.2). IHD was significantly associated with hypertension and diabetes (p = 0.001, each). From all IHD patients, 90.24% had hypertension with 13.5% had good hypertension control, 59.5% had poor hypertension control, and 27% were newly diagnosed hypertension. Similarly, 41.5% of IHD patients had diabetes mellitus with 41.5% had good glycemic control, 35.3% had poor glycemic control and 17.6% were newly diagnosed T2DM. About 11%(40) of CVD admissions had pulmonary heart disease(PHD) with over 42% of them were attributed to COPD and the remaining were due to chronic PTE and post-TB fibrosis. PHD was more common in patients who came from Addis Ababa than those coming outside of Addis Ababa but the difference was not significant (p = 0.053). Females constituted over two-third of patients diagnosed with dilated cardiomyopathy and peripartal cardiomyopathy accounted for 26.5%(p = 0.01).

Vascular diseases (VasD) were the most common cause of cardiovascular diseases next to CHF and HHD. It accounted for about a quarter of cardiovascular diagnosis and females constituted about two-third of the cases. DVT was the leading vascular cause accounting for 44%(37) followed by PTE of about 30%(25). Peripheral arterial disease (PAD) and cerebral venous thrombosis(CVT) accounted for 15.5%(13) and 9.5%(8) of vascular disease admissions, respectively. Pregnancy and related conditions such as caesarian section and puerperium (30.6%(26)); major surgery and prolonged immobilization for medical illnesses(28.2%(24)); and active cancers(11.7%(10)) were the major risk factors of venous thrombo-embolism(VTE). Hypertension and diabetes were significantly associated with PAD(p = 0.001, each). Hypertension was documented as a risk factor in about 85% of patients with PAD followed by diabetes mellitus and chronic kidney disease, each of which were implicated as a risk factor in 25% of cases of PAD. The cumulative rate of vascular diseases have significantly decreased as the age of the patients increased(p = 0.01) (Fig 1).

**Fig 2:**
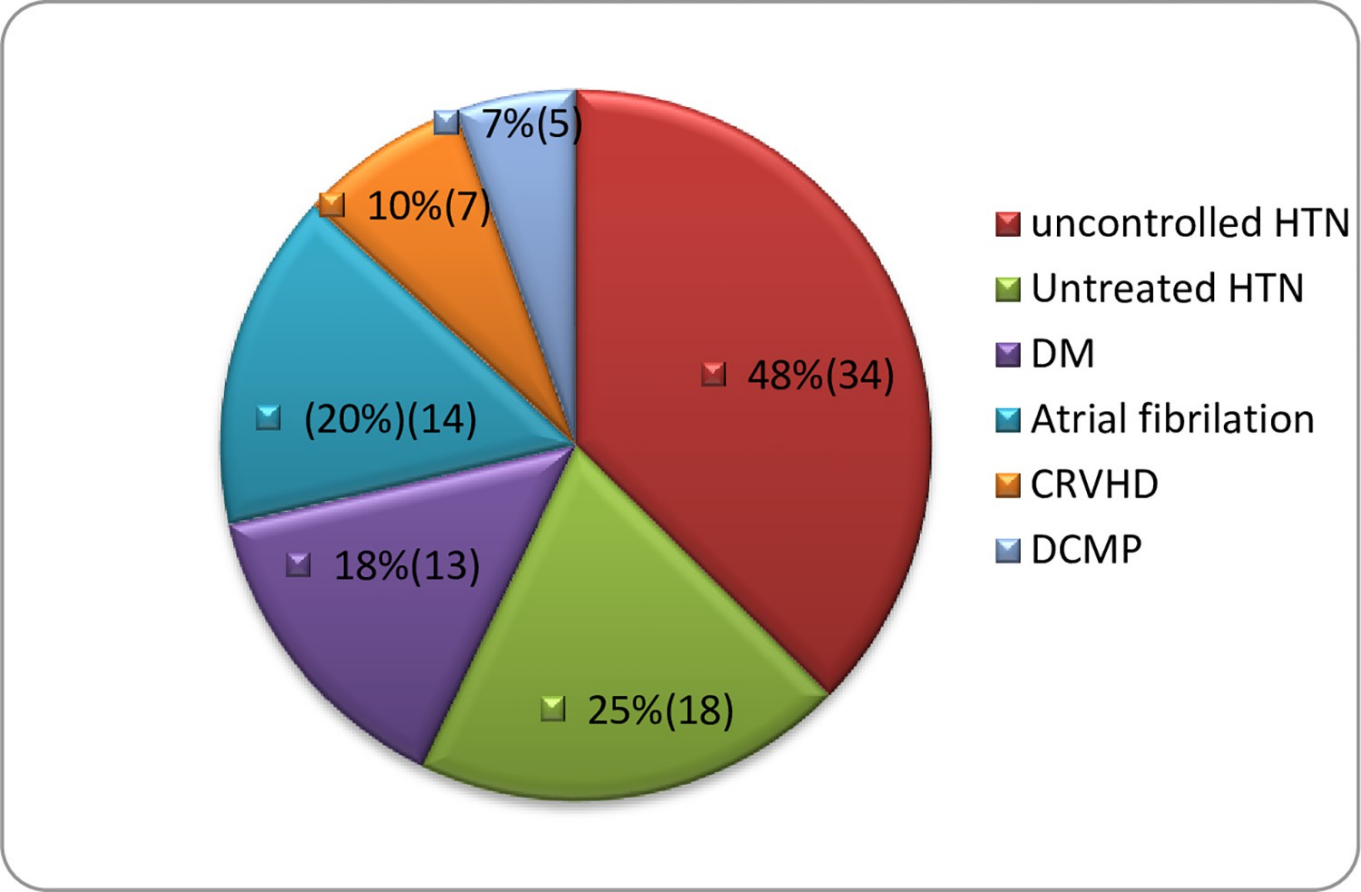
Major risk factors of stroke among admitted patients in St Paul Hospital Millennium Medical College.

Cerebrovascular accident (stroke) accounted for about 20%(71) of total annual cardiovascular admission with hemorrhagic stroke constituted about 17% and ischemic stroke for the remaining 83% of the total stroke patients. Of all ischemic stroke, cardioembolic stroke accounted for 32.2%. From all the atherothrombotic ischemic stroke groups, 7.5% had hemorrhagic transformation during the course of hospital stay. Stroke was predominant in residents of Addis Ababa than those who came outside of Addis Ababa although the difference was not statistically significant (p = 0.07) but it increased with increasing age(p = 0.001) (Fig 1). There was no variation of stroke distribution between males and females (male = 45% and female = 55%, p = 0.09). Hypertension was the leading risk factor for stroke, followed by atrial fibrillation (Fig 2). All patients with hemorrhagic stroke had hypertension and 75% of them had long standing uncontrolled hypertension whereas the remaining 25% had newly diagnosed hypertension. Similarly, 97.5% of atherothrombotic ischemic stroke(non-cardio-embolic) patients had hypertension with 61.5% of them had uncontrolled hypertension whereas about 38% had newly diagnosed hypertension after the current presentation. About one-third of atherothrombotic ischemic stroke patients had diabetes. Atrial fibrillation accounted for 73.7% of cardioembolic stroke admissions with 71.5% of CRVHD patients and 60% of DCMP patients had atrial fibrillation.

#### Hypertension and diabetes control among the cardiovascular admissions

Of all the 353 annual cardiovascular admissions, 47.6%(168) patients had history of hypertension. Hypertension was prevalent in patients from Addis Ababa than patients who came outside of Addis Ababa ((63.7%(107) Vs 36.3%(61), p = 0.001)). The minimum duration of hypertension ranged from newly diagnosed hypertension up to the maximum of 30 years. Only 20% of patients had good hypertension control (BP<140/90mmHg) with adherence to the medications and have frequent follow up whereas 46.6% of patients had poor hypertension control (BP≥140/90mmHg) who were either not adherent to or discontinued their pharmacologic therapy by themselves. About 35% of hypertensive patients were newly diagnosed during their current admission. The control of hypertension in patients from Addis Ababa was significantly better than patients coming outside of Addis Ababa (p = 0.001). The proportion of diabetes mellitus(DM) among the cardiovascular admissions was 17.6%(62) and significantly prevalent in patients from Addis Ababa than those out of Addis Abbaba ((74.1%(46) Vs 25.8%(16), p = 0.001)). The duration of DM history ranged from newly diagnosed DM up to 25 years. About 40% of DM patients had ’’good’’ DM control with mean FBS≤130mg/dl and/or A1C<7% whereas 46.8% had ’’poor’’ DM control (mean FBS>130mg/dl and/or A1C>7%). The control of diabetes in patients from Addis Ababa was significantly better than those coming outside of Addis Ababa (p = 0.001).

### Outcomes of cardiovascular disease admissions

Among patients with CVD admissions, the minimum hospital stay was one day and the maximum was 80 days with the average hospital stay was 12days. About 57%(201) of cardiovascular admissions were discharged from the hospital with improvement, 10%(36) were discharged with the same condition(most were stroke patients with dense hemiplegia and also stage-D CHF patients), and 4.5% (16) were left against medical advice. The in-hospital mortality rate among the cardiovascular admission was 24.3%(86) with the predominated immediate cause of death being sepsis with septic shock(25.6%) followed by fatal arrhythmia (19.8%), brain herniation (15%), massive PTE (14%), and cardiogenic shock (11.6%) (Table 3).

**Table 3:** Major causes of death among cardiovascular admissions in St Paul Hospital.

Causes of death	frequency	percentage
**Septic shock**	22	25.6%
**Fatal arrhythmia**	17	19.8%
**Brain herniation**	13	15%
**Massive PTE**	12	14%
**Cardiogenic shock**	10	11.6%
**Unexplained Sudden cardiac arrest**	6	7%
**Cause of death no documented**	3	3.5%
**Massive aspiration**	1	1.56%

## Discussion

The epidemic of cardiovascular disease (CVD) is a global phenomenon, and the magnitude of its burden is alarmingly increasing in the developing world including Ethiopia [6]. In the current study which involved all the annual medical admissions of 1,165 patients at St. Paul Hospital Millennium Medical College, the total cardiovascular admission constituted 30.3%. The present burden of cardiovascular causes of medical admission was in agreement with the previous studies [25]. However, the previous studies considered hypertension as a separate exclusive CVD diagnosis but in this study hypertension was considered as a CVD risk factor and hypertensive heart disease was an exclusive diagnosis as per the International Disease Classification(ICD-10) [22]. The present finding is also in tandem with the cardiovascular admission of 31% among all medical admission in the Nigerian Teaching hospital [26]. However, the current rate of CVD admission is much greater than 17.5% of CVD admission in Asella Referal Hospital [27]. This difference might be due to large proportion of urban residence in our study dominated by cardiovascular risk factors including hypertension and diabetes unlike the former area where infectious causes of admission accounted for close to 50% [27].

Most of the CVD patients (60%) were admitted with the clinical diagnosis of advanced congestive heart failure (CHF) (NYHA class III and IV). This high rate of CHF in the current study is in agreement with other Ethiopian studies [25,28]. Surprisingly, apart from hypertension and pneumonia, over a quarter of CHF patients had associated history of drug discontinuation as a precipitating factor eventually worsening their heart failure condition. Therefore, healthcare providers should also equally focus on health education including adherence to the medical therapy in addition to prescribing the pharmacologic agents based on the appropriate diagnosis.

Hypertensive heart disease (HHD) was the second predominant diagnosis constituting for 41% of overall cardiovascular admission and also the leading cause of heart failure followed by rheumatic valvular heart disease(RVHD) which constituted for about 18% of all CVD. In most of the previous studies involving both inpatient and outpatient follow up clinics, VHD was considered to be the leading cause of CVD in general and cardiac diseases in particular [17,18,25,27–29]. This increased burden of HHD shown in the current study, however, is consistent with other recent studies in Ethiopia [18]. The high proportion of hypertension (about 48%) among our CVD patients also supports the high rate of HHD. Compared to rural dwellers, HHD in the current study was predominant in urban residents(p = 0.01). It may be partly due to an increasing urbanization and westernization of diet in the present metropolitan area coupled with better health care availability allaying the risk factors for rheumatic valvular heart disease (RVHD). RVHD in the current study is dominant in patients coming from rural area than the residents of Addis Ababa(p = 0.001). Rural predominance of RVHD is consistent with previous studies [16–18]. This could partly be justified by the early treatment of upper respiratory tract infection and better access to health care in urban residents. RVHD in the present study was also predominant in females (p = 0.001) and it is in agreement with previous studies [16,17]. Ischemic heart disease (IHD) and dilated cardiomyopathy(DCMP) were other causes of cardiac diseases constituting for 12.2%, and 9.6% of annual CVD admissions, respectively and their burden increased with increasing age(p = 0.01). These findings are in agreement with other national studies [16,18,28]. The tall of ischemic heart disease is significant when the current rate of 12,200 per 100,000 is compared with the data of 7,400 per 100,000 in 2014 [15]. The risk of IHD was associated with Hypertension and Diabetes mellitus (p = 0.001, each) and is in agreement with similar studies [15].

Cerebrovascular accident (CVA) or stroke accounted for 20% of annual CVD admissions with ischemic stroke being the predominant sub-type(83%), and hemorrhagic stroke constituted the remaining 17%. This finding is consistent with the well-established global data and some of Ethiopian studies where Ischemic stroke is the dominant sub-type [15,30–32]. However, the current finding is not in agreement with some of the Ethiopian data in which hemorrhagic stroke was reported to be the dominant subtype [33–35]. The exaggerated hemorrhagic stroke compared to ischemic stroke which was reported in a number of studies in Ethiopia mandates further scrutiny. In this study, hypertension was present in 74% of patients with stroke. All hemorrhagic stroke patients had hypertension and 75% of them had long standing uncontrolled hypertension whereas the remaining 25% had newly diagnosed hypertension. This finding is in agreement with other similar studies [30,33,36]. Since one-third of our ischemic stroke patients had pre-existing atrial fibrillation (AFib) and AFib significantly raises the likelihood of stroke, effective preventive therapy is critical and should be one of the key management priorities [14]. Furthermore, 97.5% of atherothrombotic(non-cardioembolic) ischemic stroke patients had hypertension as a major risk factor where 61% of them had uncontrolled hypertension and 38% of them had newly diagnosed or untreated hypertension. This finding is also in conformity with other studies [30,33,36]. Therefore, poor screening, poor follow up and adherence against CVD risk factors including hypertension, makes it imperative to re-evaluate our management practices, so as to establish a consolidated approach involving the relevant stakeholders. For countries of poor economy including ours, prevention is much cheaper and profitable than the complex and costly managements of the subsequent CVD diseases and their complications. Therefore, timely screening for the cardiovascular risk factors as well as early identification of complications utilizing various approaches including technology of telemedicine could be an efficient, and cost effective method [37].

Hypertension, in the current study, was found to be the dominant risk factor for cardiovascular diseases where about 48% of CVD patients had hypertension. However, this finding is higher than some of the previous reports of hypertension burden among the CVD patients in Ethiopia [15,16,17,25,38] but much lower than the 62% as shown in the study conducted in Gondar Referral Hospital [18]. Our finding is also consistent with the overall increasing trends of atherosclerotic CVD burden in the recent decades with hypertension being a significant risk factor [18,39]. The worrisome finding was that only 20% of our patients with longstanding hypertension had good hypertension control whereas about 47% of patients had poor hypertension control who were either not adherent to or discontinued their antihypertensive therapies by themselves. Furthermore, 35.2% of hypertensive patients were newly diagnosed or told to have raised blood pressure in the past but not on therapy or follow up. This poor hypertension control is consistent with other studies [40,41] and warrants timely intervention. Similarly, from all CVD patients with long standing diabetes mellitus, only 40.3% had good glycemic control whereas 46.8% had poor glycemic control and poor follow up. About 13% of diabetes was newly diagnosed type-2 diabetes after they were admitted for the current cardiovascular illness. These findings are consistent with other studies in Ethiopia [42,43] but much better than the 20% of good glycemic control at Tikur Anbessa Specialized Hospital [24]. Despite the rising cost of newer cardiovascular drugs are challenging the drug adherence in the settings with poor economies, some of the older, cheaper and effective agents can still be easily available to serve as a backbone and drug of first choice [44] so as to improve the drug adherence.

Vascular diseases including deep venous thrombosis (DVT), pulmonary thromboembolism (PTE), peripheral arterial disease (PAD), and cerebral venous thrombosis (CVT) were common in the present study area and all together constituted about 24% of all the annual CVD admissions. DVT was the leading cause of vascular admission (44%), followed by PTE(30%), PAD(15.5%) and CVT (9.5%), respectively. The current vascular disease burden is much higher than the previous cardiovascular spectrum studies [15,16,18] which involved the outpatients, unlike the admitted patients who have higher risk factors [45,46]. Major risk contributors of venous thromboembolism (VTE) were pregnancy and its related conditions such as puerperium and cesarean section (30.6%), major surgery and prolonged immobilization for medical illnesses (28.2%), and active cancers (11.7%). The higher burden of DVT, younger age and female predominance, major risks (pregnancy and puerperium, prolonged immobility and malignancy) documented in the current study are consistent with previous studies in Addis Ababa Hospitals [45,46]. The high rate of VTE, including massive PTE as one of the major immediate cause of death (Table 3), may be partly due to lack of comprehensive guideline based venous thromboembolism (VTE) prophylaxis consistent with the degree of risk factors. The Ethiopian custom of prolonged bed rest (immobility) during puerperium might have also exacerbated the existing procoagulant risks. Hypertension was a risk factor in about 85% of patients with PAD followed by diabetes mellitus (25%). This is consistent with the association of uncontrolled hypertension and diabetes with atherosclerotic CVD diseases including PAD [38]. Therefore, such findings mandate comprehensive approach to develop proper CVD risk assessment and disease control.

The mean duration of hospital stay in the current study was 12days with the minimum hospital stay was one day and the maximum was 80 days. This result is comparable with the average of 12.33days of hospital stay for medical admission [25] and 11.14days for stroke admissions at St. Paul hospital [34] but less than the 14.4days of total hospital stay at Asella referral hospital [27]. There were 57% of CVD patients discharged with improvement and in-hospital mortality rate was 24.3%. These findings are comparable to the previous St. Paul hospital reports of in-hospital death rate of 24.2% and discharge rate with improvement of 66% among the medical admissions [25]. Despite the current mortality rate is lower than the 25% documented among the cardiovascular admissions in Addis Ababa [28] and the 30% mortality rate among stroke admissions in St. Paul Teaching hospital [34], it is higher than the 12.2% among cardiovascular admissions in Nigeria [26]. Sepsis with septic shock was the leading immediate cause of death in about a quarter of deaths among CVD admissions, followed by fatal arrhythmia, brain herniation, massive PTE and cardiogenic shock in 20%, 15% and 14%, respectively. Consistent with our study, infectious disease was also the leading cause of mortality among medical admissions studied previously [25,27]. Therefore, comprehensive work is needed to improve impatient medical care in addition to CVD risk assessment, and disease control.

### Limitations

Due to the retrospective nature of the study, it has a limitation of including additional cardiovascular risk factors including smoking history, obesity, and physical inactivity. Since it was also a hospital based study with limited sample size, it lacks the accurate representation of the actual burden of cardiovascular diseases in the wider community. Furthermore, incomplete documentation is common in the retrospective studies. To avoid the sampling bias, we have incorporated the entire annual medical admissions in the study.

### Conclusion

In conclusion, cardiovascular diseases are common in the study area causing significant morbidity and mortality. Most cardiovascular diseases have preceding unaddressed but easily modifiable risk factors, controlling which could have prevented or delayed the progression of the disease. Therefore, concerted effort is required from all stake-holders in terms of risk reduction as well as follow-up optimization to delay or if possible to prevent cardiovascular disease development, disease progression and subsequent complications. Strategies should also be designed to increase public awareness regarding cardiovascular risk reduction, drug adherence, and possible complications.
